# Biodegradable Preformed Particle Gel (PPG) Made of Natural Chitosan Material for Water Shut-Off Application

**DOI:** 10.3390/polym15081961

**Published:** 2023-04-20

**Authors:** Reem Elaf, Ahmed Ben Ali, Mohammed Saad, Ibnelwaleed A. Hussein, Hassan Nimir, Baojun Bai

**Affiliations:** 1 Gas Processing Center, College of Engineering, Qatar University, Doha P.O. Box 2713, Qatar; 2 Department of Chemical Engineering, College of Engineering, Qatar University, Doha P.O. Box 2713, Qatar; 3 Department of Chemistry and Earth Sciences, College of Arts and Science, Qatar University, Doha P.O. Box 2713, Qatar; 4 Department of Geosciences and Geological and Petroleum Engineering, Missouri University of Science and Technology, Rolla, MO 65409, USA

**Keywords:** preformed particle gel, water shut-off, chitosan, environmentally safe, swelling, mechanical strength, thermal stability

## Abstract

Oil and gas extraction frequently produces substantial volumes of produced water, leading to several mechanical and environmental issues. Several methods have been applied over decades, including chemical processes such as in-situ crosslinked polymer gel and preformed particle gel, which are the most effective nowadays. This study developed a green and biodegradable PPG made of PAM and chitosan as a blocking agent for water shutoff, which will contribute to combating the toxicity of several commercially used PPGs. The applicability of chitosan to act as a crosslinker has been confirmed by FTIR spectroscopy and observed by scanning electron microscopy. Extensive swelling capacity measurements and rheological experiments were performed to examine the optimal formulation of PAM/Cs based on several PAM and chitosan concentrations and the effects of typical reservoir conditions, such as salinity, temperature, and pH. The optimum concentrations of PAM with 0.5 wt% chitosan were between 5–9 wt%, while the optimum chitosan amount with 6.5 wt% PAM was in the 0.25–0.5 wt% range, as these concentrations can produce PPGs with high swellability and sufficient strength. The swelling capacity of PAM/Cs is lower in high saline water (HSW) with a TDS of 67.2976 g/L compared with fresh water, which is related to the osmotic pressure gradient between the swelling medium and the PPG. The swelling capacity in freshwater was up to 80.37 g/g, while it is 18.73 g/g in HSW. The storage moduli were higher in HSW than freshwater, with ranges of 1695–5000 Pa and 2053–5989 Pa, respectively. The storage modulus of PAM/Cs samples was higher in a neutral medium (pH = 6), where the fluctuation behavior in different pH conditions is related to electrostatic repulsions and hydrogen bond formation. The increase in swelling capacity caused by the progressive increment in temperature is associated with the amide group’s hydrolysis to carboxylate groups. The sizes of the swollen particles are controllable since they are designed to be 0.63–1.62 mm in DIW and 0.86–1.00 mm in HSW. PAM/Cs showed promising swelling and rheological characteristics while demonstrating long-term thermal and hydrolytic stability in high-temperature and high-salinity conditions.

## 1. Introduction

Unwanted excess water during the extraction process of oil and gas is considered one of the most severe problems facing the petroleum industry today and results in leaving a significant quantity of unrecoverable hydrocarbons in the reservoir. According to the latest statistics, 75 million barrels of oil and 210 million barrels of produced water are generated daily worldwide [1]. The produced water consists mainly of toxic contaminants, including amounts of workover chemicals added during the operation of the well. For this reason, it causes increased corrosion and degradation rates in production facilities, emulsion formation, scale deposition, and, most importantly, shortens the well’s lifetime and reduces oil recoveries [2]. Consequently, the petroleum industry faces several costly wastewater challenges where handling and managing this water become unfeasible.

Considerable efforts to address water shut-off technologies have been executed in the past few decades to reduce this phenomenon as much as possible. One technology uses cement, plugs, and packers to mechanically shut-off the water [3]. An alternative, more effective, and widely practiced method is chemically managing the overflow of unwanted water, such as by applying in-situ crosslinked polymer gels. The in-situ gel is the injection of a low-viscosity solution of polymer/crosslinker to form a gel at a reservoir environment with appropriate pH and temperature [3]. The most economically attractive polymer commonly used in the in-situ application is polyacrylamide (PAM). Various organic and inorganic crosslinkers can provide a high-strength stable gel with PAM, such as polyethyleneimine, chromium, aluminum, zirconium, phenol/formaldehyde, and others [4].

In-situ crosslinked gels have a wide range of stability at reservoir temperature and salinity conditions. However, the gelation process is very unpredictable, and the gel quality is tough to control because the gelation system always has problems with chromatographic separation, shear-thinning, and dilution by the formation water during their flow into the reservoir [5]. The in-situ gel treatment’s effectiveness is further constrained by the ease with which the polymer before gelation can be thermally degraded during transport into a reservoir with high salinity and high temperature conditions [5].

A new chemical approach has been introduced to tackle water problems: preformed particle gels (PPGs) injection. PPGs are defined as crosslinked polymeric particles that are dispersible in water used in oil and gas reservoirs. Unlike in-situ gelation, PPGs are prepared and crosslinked in surface facilities, where the crosslinked gel network is dried and crushed into small sizes depending on the target application. The working principle of PPGs is that they swell in water, leading to an increase in size with time where it is designed to reach the maximum swellability at the designated area. Polymers with hydrophilic functional groups, such as PAM, are usually used, leading the PPGs to swell from a few times to more than 200 times their original size in an aqueous environment [6]. PPGs can be applied to plug near-wellbore fractures and for in-depth profile modification since small-size PPGs can penetrate deeper into the porous media [7].

Particle gel treatment has distinctive advantages over ordinary in-situ gels. For instance, they overcome the variations in gelation and uncontrollable crosslinking times because of shear degradation and gelant alterations brought on by interaction with reservoir fluids and minerals. PPGs are multi-sized and possess proper selectivity properties that can favorably flow into channels or fractures while avoiding penetration into low-permeable oil-saturated zones, hence mitigating formation damage of unswept zones by gel [8]. In addition, they are stable in saline formation water and most of the reservoir mineralogies, strong and size-controllable, and environment-friendly [7]. According to Seright’s (2003) study, PPGs are more effective in achieving water conformance than in-situ gels [9]. Another finding by Heidari et al. (2019) was that PPGs outperformed in-situ gels in terms of oil recovery [10]. Seright (2001) and Seright et al. (2003) approved that PPG had superior placement than in-situ gel and could successfully prevent gel damage on low-permeability, unswept oil zones [11,12].

PPG has been the subject of a great deal of research in recent years, with the majority of these studies concentrating on the effects of PPG composition (monomer/polymer and crosslinker concentrations) and the environmental parameters (such as temperature, salinity, and pH) on swollen PPG strength and swelling capacity [13,14,15,16,17]. A few studies have taken long-term thermal stability and aging tests into account [18,19]; others included the particle size study of the swollen PPGs to place the appropriate size in the designated fracture zone [20,21,22]. Therefore, it is vital to consider all aspects above when creating innovative PPG formulations.

Many synthetic polymers have been used in formulating PPGs. Nevertheless, polyacrylamide, polyacrylic acid, and the co-polymer combining are the most abundant. Typically, organic crosslinkers such as N, N-methylene bis-acrylamide [18,23,24] or inorganic crosslinkers [14,25] are utilized in the process of PPG’s production. Some PPGs share formulations or crosslinkers with those used in the in-situ gels, such as polyethylenimine [19], chromium acetate [25], and aluminum nitrate [14], which indicates that the formulations of the current in-situ gels might also develop PPGs for water shut-off treatment, having the advantage of being PPG.

Synthetic polymers are made from petrochemical derivatives. They are used and dumped into the environment as non-degradable waste, where their accumulation seriously destroys the planet’s natural environment [26]. According to El-Karsani et al. (2014), some crosslinkers, such as Chromium acetate and Polyethyleneimine (PEI), possess some toxicity to marine life [3]. According to the Oslo and Paris Commission (OSPAR), offshore chemicals’ overall impact on the marine ecosystem must be reduced. Accordingly, developing green polymeric materials that are biodegradable, non-toxic, and environmentally safe becomes necessary.

Recently, our team has verified using chitosan as a green crosslinker to replace the PEI crosslinker and produce a stable in-situ polymeric gel in high-temperature and low-acidity reservoirs [27]. Chitosan is made from chitin, which is the second-most prevalent polymer in nature. In addition to being a biodegradable, non-toxic, and renewable substance [27]. Chitosan can operate as a hydrogel because it contains amino and hydroxyl groups, which are hydrophilic functional groups [28]. This wide range of qualities makes it a good choice for developing an environment-safe PPG and reducing the need for synthetic polymers.

This paper proposes a novel green PPG to continue exploiting chitosan’s qualities in water shut-off treatment and contribute to broadening the scope of natural materials in oil field applications worldwide. Therefore, this study aims first to conduct a preliminary screening of a range of base-polymer and crosslinker concentrations to identify the optimum PPGs that provide high mechanical strength and swelling capacity. Secondly, to analyze the compositions and chemical structures of the gel particles using the infrared spectroscopy analysis method. Moreover, examine how the PPG’s performance is affected by salinity, pH, temperature, and aging. Finally, investigate the swollen PPG’s size to define the appropriate placement zone.

## 2. Materials, Procedures, and Methods

### 2.1. Materials

A chitosan sample with an 88% degree of deacetylation and a low average molecular weight was acquired from ChitoLytic, Toronto, ON, Canada. SNF Floerger, Andrézieux, France, supplied the polyacrylamide (PAM) samples with a molecular weight of 700,000 Da. Other chemicals, such as Acetic Acid Glacial solution (CH_3_CO_2_H), are acquired from the Research Lab Fine Chem Industries, Mumbai, India.

### 2.2. Experimental Procedures

The PPGs of chitosan and PAM were made in phases using various ratios of chitosan (0.13–2%) and PAM (3.5–9%). Firstly, specific quantities of polyacrylamide liquid are diluted with deionized water, and then the pH of the solution is lowered to below 6.5 by adding 1 wt% of acetic acid. The chitosan powder was gradually added to the solution at a predetermined concentration to create a well-mixed solution and to give the flocculated and agglomerated chitosan particles time to de-flocculate and dissolve. The created solutions were then transferred into screw-cap bottles and placed in an oil bath for 24 h at 80 °C to undergo a transamination reaction and form cross-linked gels. The gel samples were then removed, cut into small pieces, and stored in an oven at 80 °C for another 24 h to obtain a completely dried polymer gel. The dried structures are finally crushed, ground, and sieved into a particle size range of 250–500 µm, creating semi-uniform PPGs.

### 2.3. Methods

#### 2.3.1. Fourier Transform Infrared Spectroscopy (FTIR)

Fourier transform infrared spectroscopy (FTIR) was carried out using Perkin Elmer Spectrum (Llantrisant, UK) with a KBr disc for direct compression, with 16 scans on average at 4.0 cm^−1^ resolution in the wave number range of 400–4000 cm^−1^ on dried powders of chitosan and chitosan/PAM to confirm the chemical structure and the compositions of the generated PPG.

#### 2.3.2. Scanning Electron Microscope (SEM)

Scanning electron microscopy (SEM) was used to visualize the morphological structures of the swollen PPGs. Small pieces of swollen PPG were put through three freeze-drying stages for 48 h using the Labconco FreeZone 12 machine (Kansas City, MO, USA), as the conventional SEM does not accept any humid samples. The swollen PPGs were frozen at −50 °C at 0.6 °C/min in the freeze-dryer chamber. Furthermore, they were sublimated at a temperature of −15 °C and a total gas pressure of 18 Pa. Afterward, the samples were dried at room temperature of 25 °C under a pressure of 10 Pa to optimize the residual moisture content. The FEI Nova Nano SEM 450 microscope (Eindhoven, The Netherlands) was used to conduct SEM testing, after which the samples were sprayed and coated with gold.

#### 2.3.3. Swelling Behavior

The swelling ratio was measured using the gravimetric technique, which requires soaking 0.5 g of dry PAM/Cs PPGs in 100 mL of distilled water (DIW) or brine solution of 1% NaCl, saline water (SW), and high saline water (HSW) for set periods at a prespecified temperature (25–100 °C) or pH. The considered particle size of the dried PPGs in this study is in the range of 250–500 µm. The PPG samples were left soaking in water and stirred for 24 h to ensure that the water had contacted all the particles and that they had reached their maximum swelling capacity. The swollen PPGs were detached from the residual solution using filter paper and weighted to calculate the swelling ratio (g/g) using Equation (1).

(1) $$\mathrm{SR}\;\left(g/g\right)=\frac{W_{s}-W_{d}}{W_{d}}$$

where $W_{s}$ is the weight after swelling, and $W_{d}$ is the weight of dry PPG before swelling.

Table 1 illustrates the total dissolved salts (TDS) of the SW and HSW employed in this study, where pure salts are dissolved in deionized water on the laboratory scale. The specified TDS of the HSW and SW, which are 67.3 g/L and 33.6 g/L, respectively, represent the salinity levels prevalent in oil reserves in Gulf nations.

All the experiments were duplicated to ensure minimal errors, and the average values were considered.

#### 2.3.4. Rheology Measurement

An Anton Paar MCR 302 rheometer was used to investigate the PPG samples’ storage modules (G′). The storage modulus (G′) indicates the maximum stretch or deformation that the gel can withstand before it breaks down [31], reflecting its ultimate strength. The storage modulus and linear viscoelastic region (LVR) of the swollen PPGs are investigated using a frequency sweep test with a frequency range of 0.1 to 100 Hz, employing parallel plate geometry with a 25 mm diameter and 2 mm spacing. The storage modulus values at 10 Hz were selected to compare different gel samples, ensuring all testing was within the LVR at a specified strain of 10%. Before undergoing rheological testing, the PPGs were prepared under predefined temperature, pH, and salinity conditions and given adequate time to attain their maximum swelling capacity. Each experiment was repeated twice to ensure an error of less than 5%.

#### 2.3.5. Particle Size

PPGs come in a variety of particle sizes depending on their intended use. In this work, dried gels are crushed and sieved down to a size equivalent to a sieve size of 250–500 µm to create micro-sized PPGs. Moreover, it is vital to consider the particle size of the swollen PPGs to specify the diameter of the pore throat or fracture that may be sealed up. Therefore, Equation (2) was employed to evaluate the swollen particle size in the presence of all other parameters, such as the initial dried diameter and swelling ratio.

(2) $$D_{t}=d_{0}\times Q^{1/3}$$

where $D_{t}$ is the average particle size at swelling time t, $d_{0}$ is the PPG’s initial average particle size and Q is the swelling ratio.

## 3. Results and Discussion

### 3.1. FTIR Spectroscopy Characterization

**Figure 1:**
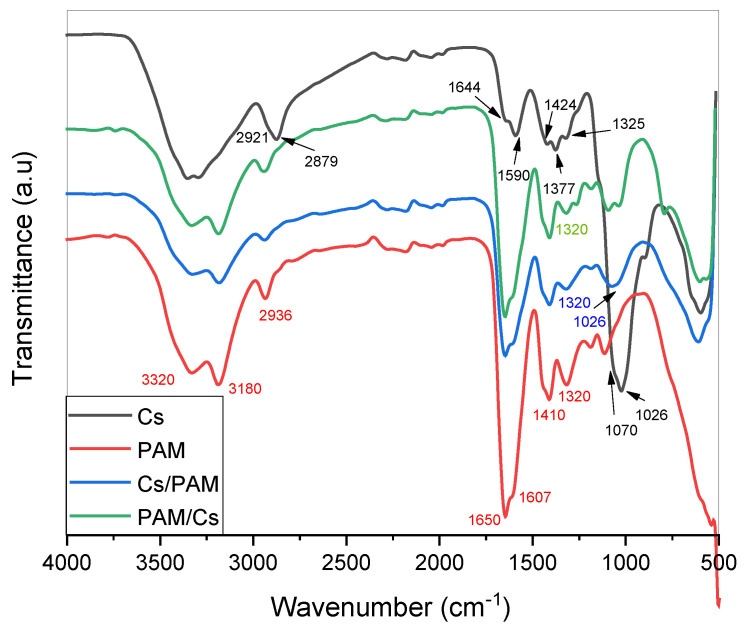
The FTIR spectra of Cs, PAM, Cs/PAM and PAM/Cs.

Figure 1 depicts the FTIR spectra of PAM, Cs, Cs/PAM, and PAM/Cs. For pure chitosan (Cs), the spectra display a broad band in the region 3200–3450 cm^−1^, related to N–H and O–H stretching as well as hydrogen bonds in the polysaccharide. The C–H symmetric and asymmetric stretching appeared at around 2921 and 2879 cm^−1^, respectively. The peaks at 1644 cm^−1^ and 1325 cm^−1^ correspond to the C=O and C–N stretching groups. The N–H bending can be seen at 1590 cm^−1^. The CH_2_ bending and CH_3_ symmetrical deformations were verified by the appearance of bands at around 1424 and 1377 cm^−1^, respectively. The characteristic peaks at 1070 and 1026 cm^−1^ are due to the C–O stretching and O–H bending, respectively.

Regarding the FTIR spectra of PAM, the characteristic peaks at 3180 cm^−1^ and 3320 cm^−1^ are attributed to symmetric and asymmetric stretching of the N–H bond, respectively. Vibration bands at 1650 cm^−1^ and 1607 cm^−1^ are attributed to C=O stretching and NH bending, while bands at 1410 cm^−1^ and 1320 cm^−1^ are attributed to C–H bending and C–N stretching, respectively.

The FTIR spectra of Cs/PAM and PAM/Cs show a similarity in the PAM spectrum, where the prepared material contains more PAM than Cs. The functional groups in both polymers have been absorbed in the same region since different vibrational modes of N–H, C–N, and C=O bonds of amines and amides groups appear in similar ranges (in the fingerprint region of 1700–1100 cm^−1^). However, the interaction between Cs and PAM is demonstrated by the appearance of characteristic Cs peaks in region 1026 cm^−1^, as this absorption band is absent in the spectrum of PAM. In addition, the decreased intensity of N–H peaks in the 3000–3350 cm^−1^ region and C–N at 1320 cm^−1^ is another indication of PAM/Cs interaction.

**Figure 2:**
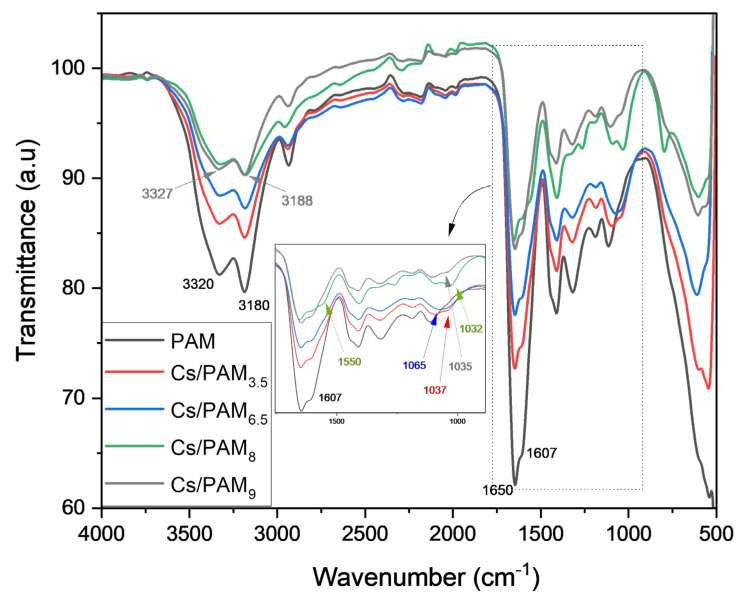
The FTIR spectra of Cs/PAM at various PAM proportions and constant ratio of Cs.

**Figure 3:**
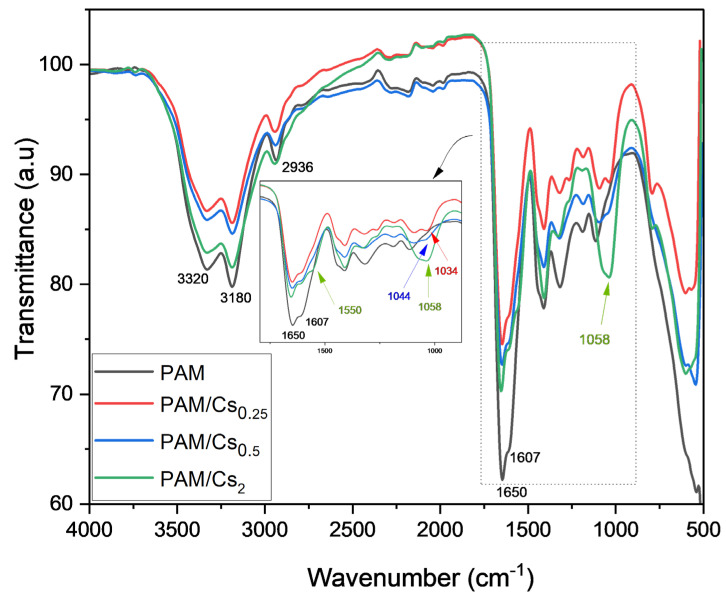
The FTIR spectra of PAM/Cs at various Cs proportions and constant ratio of PAM.

**Figure 4:**
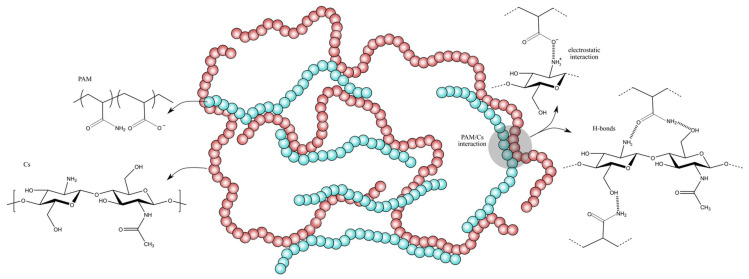
Proposed structure PAM/Cs with all possible interactions between PAM and Cs.

As shown in Figure 2 and Figure 3, the extent of the decrease in N–H bands at 3180 cm^−1^ and 3320 cm^−1^ is related to the Cs and PAM ratios in the mixture, indicating that hydrogen bonds formed between amide groups in PAM and hydroxyl and amine groups in Cs (Figure 4). A comparable explanation was given by Xiao et al. [32] and Li et al. [33] for PAM/Cs hydrogels. The peak of the N–H band shifted based on the PAM proportion in the mixture, with the maximum shift for Cs/PAM_9_ from 3180 cm^−1^ and 3320 cm^−1^ to 3188 cm^−1^ and 3327 cm^−1^, respectively. Similar findings were observed for the hydrogel-based PAM/carboxymethyl cellulose/calcium system [34] and explained by creating H-bonds between the amide and hydroxyl groups. This hypothesis is supported by the shifting of O–H bending from 1026 cm^−1^ to 1029, 1032, 1034, 1035, and 1037 cm^−1^ for Cs/PAM_8_, PAM/Cs_0.25_, Cs/PAM_9_, and Cs/PAM_3.5_, respectively. The overlapping C–O stretching with O–H banding for Cs/PAM_6.5_ and PAM/Cs_2_ resulted in broadband appearing at 1065 cm^−1^ and 1058 cm^−1^, respectively. The intensity of the C–N stretching also decreased, supporting the amide group’s involvement in the H-bond.

In the case of PAM/Cs_2_ and Cs/PAM_8_, the appearance of a new peak at 1550 cm^−1^ is related to asymmetric COO^−^ stretching, which resulted from the thermal hydrolysis of the amide groups of PAM, where this band should appear at 1560 cm^−1^, as reported by the previous study [35]. The electrostatic interaction with protonated amine groups in Cs may cause this shifting behavior. Figure 4 represents the proposed structure of PAM/Cs with all possible interactions.

### 3.2. Surface Morphology of PAM/CS PPG

**Figure 5:**
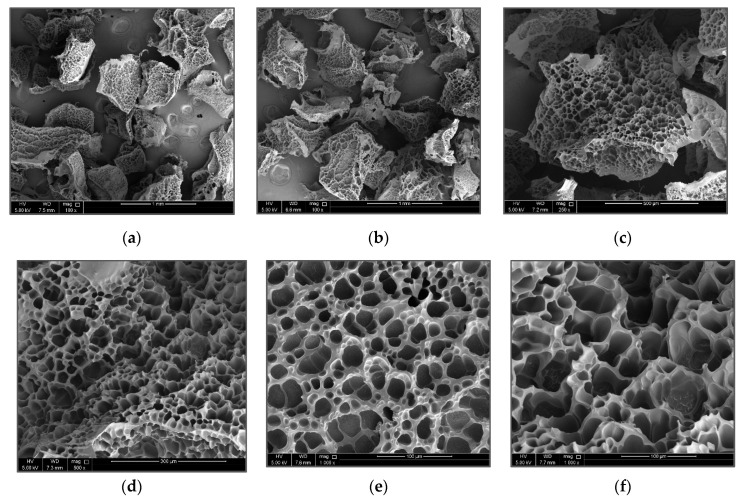
SEM images of swelled PAM/Cs PPG morphology with different magnifications (**a**,**b**) 100×; (**c**) 250×; (**d**) 500×; and (**e**,**f**) 1000×.

The SEM technique was used to visualize the morphology of swollen PPG of PAM/Cs with various magnifications, as seen in Figure 5. The pictures show that, as a crosslinker, chitosan has created a 3D network structure with an open and interconnected pore distribution. Therefore, it is evident that chitosan can build bridges between PAM chains, creating connected cages similar to a honeybee hive for water holding and storage. These uniform cages can provide the 3D network structure of swollen PPG with excellent stability. Moreover, the high porosity of the PAM/Cs hydrogel structure, as indicated in Figure 5, is crucial because it allows for more contact area with the swelling medium and facilitates water diffusion, resulting in a higher influx of water into the polymeric matrix and a higher swelling capacity [36].

### 3.3. Factors Affecting PPG Performance

#### 3.3.1. Chitosan & PAM Concentration Effect

**Figure 6:**
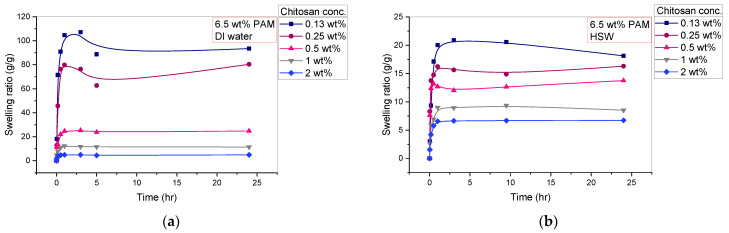
The effect of chitosan crosslinker concentration (0.13–2 wt%) on the swelling kinetics in different solution of: (**a**) DIW; and (**b**) HSW.

**Figure 7:**
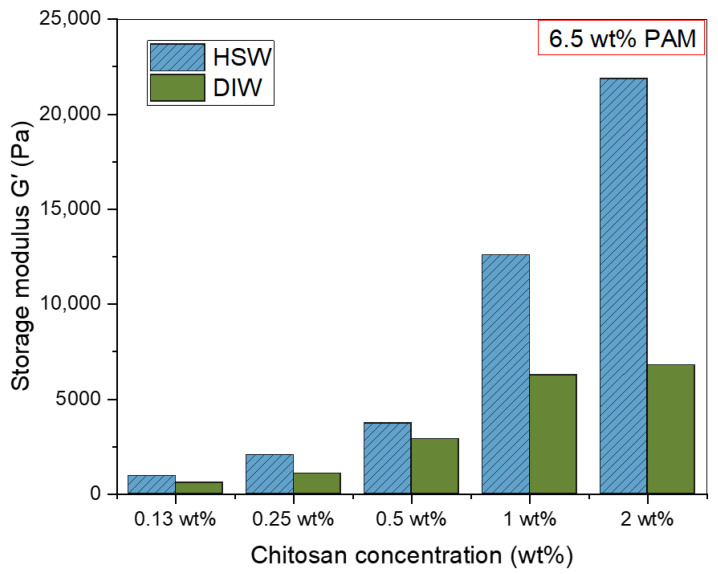
The effect of chitosan crosslinker concentration (0.13–2 wt%) on storage modulus (G’) in DIW and HSW.

The crucial factors favoring one PPG sample over another are the highest swelling capacity and strength with sufficient deformation resistance. Therefore, the swelling kinetics, swelling capacity, and mechanical strength aspects of various PPG samples, including different chitosan concentrations (0.13 wt%, 0.25 wt%, 0.5 wt%, 1 wt%, and 2 wt%) with a fixed polyacrylamide (PAM) amount of 6.5 wt%, were examined to define the function of the chitosan as a crosslinker and to choose the best chitosan concentration based on these aspects. The study was made in two swelling media, distilled water (DIW) and high salinity water (HSW), as shown in Figure 6 and Figure 7. Figure 6 depicts the profiles of the swelling ratios of the PPGs in DIW and HSW as a function of swelling time. These profiles show a rapid increase in the first 30 min, followed by a modest decrease and flattening over the next few hours. All the PPG samples reach their maximum equilibrium swelling capacity after approximately 30 min to 1 h, suggesting that PPGs’ behavior in DIW and HSW is the same but with different swelling ratios, as will be discussed in the next section of the salinity effect. The order of the absorbency of the PAM_6.5%_/Cs PPGs based on varying chitosan concentrations is PAM/Cs_0.13%_ > PAM/Cs_0.25%_ > PAM/Cs_0.5%_ > PAM/Cs_1%_ > PAM/Cs_2%_. However, as indicated in Figure 7, the mechanical strength is ordered oppositely, where PAM/Cs_2%_ has the highest storage modulus, followed by PAM/Cs_1%_, PAM/Cs_0.5%_, PAM/Cs_0.25%_, and PAM/Cs_0.13%_. Numerous systems in the literature have demonstrated the same trend of an inverse relationship between the PPG’s strength and its ability to absorb water [14,31,37]. For instance, increasing the chitosan concentration from 0.13 wt% to 2 wt% has reduced the swelling ratio from 107.11 g/g to 4.97 g/g but increased the storage modulus from 584.74 Pa to 6775.7 Pa, respectively. This relationship could be explained by the fact that increasing the chitosan concentration results in 3D polymer gel network structures that are rigid, denser, and have tiny pore sizes, which create steric hindrance toward the PPG’s expansion. As a result, it prevents significant amounts of water from entering the PPG’s network and reduces their water uptake. In addition, when the chitosan concentration increases, more hydrophilic amide groups (–CONH–) in the polymer chains of PAM, which are mainly responsible for water holding, will participate in the gelation reaction with the large number of amine groups in chitosan, resulting in lower hydrophilicity and swelling capacity [13]. Given that a concentration of 0.13 wt% produces a weak gel in DIW and that concentrations of 1 wt% and 2 wt% have poor swelling capacities, the optimum crosslinker concentrations with 6.5 wt% PAM are thus in the range of 0.25–0.5 wt%.

**Figure 8:**
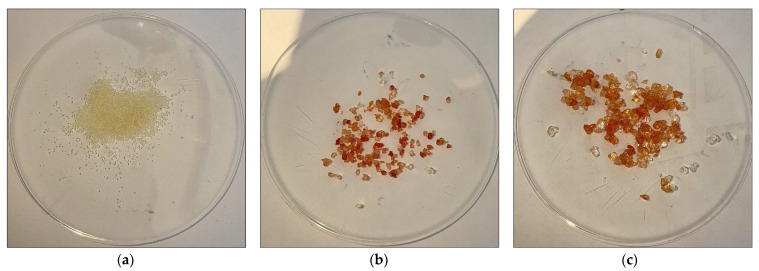
PPGs swelled in DIW for 24 h (**a**) dry PPG before being swelled, (**b**) swelled PAM_3.5%_/Cs_0.5%_, (**c**) swelled PAM_9%_/Cs_0.5%_.

**Figure 9:**
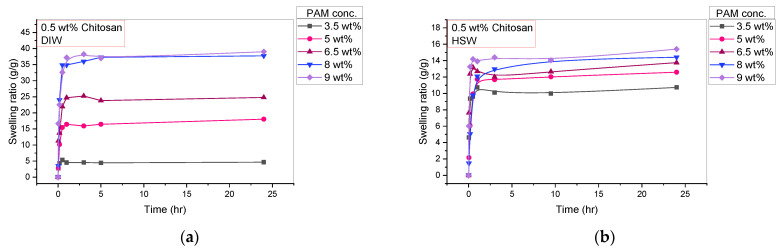
The effect of PAM concentration (3.5–9 wt%) on the swelling kinetics in different solution of: (**a**) DIW; and (**b**) HSW.

**Figure 10:**
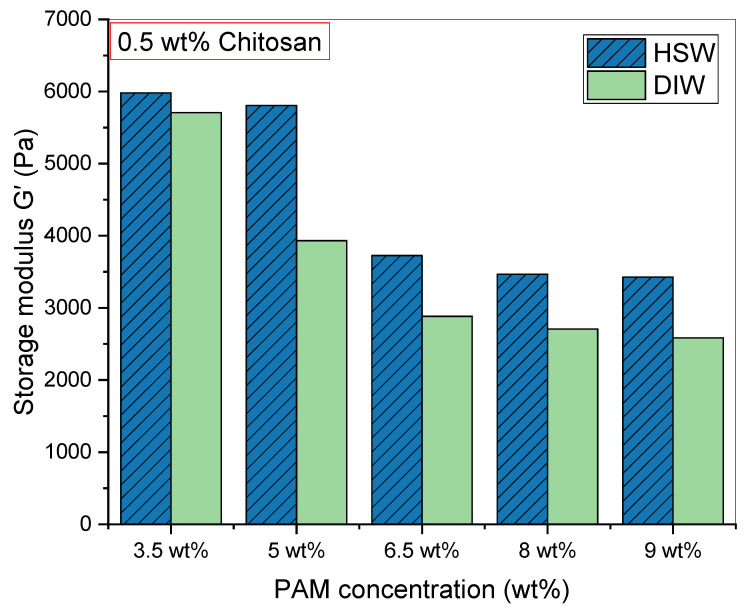
The effect of PAM concentration (3.5–9 wt%) on storage modulus (G’) in DIW and HSW.

Moving on to the investigation of the PAM concentration effect on the swelling and rheological properties of PAM/Cs PPG, five samples of different PAM concentrations (3.5 wt%, 5 wt%, 6.5 wt%, 8 wt%, and 9 wt%) with a fixed 0.5 wt% of chitosan were prepared and soaked in two solutions of DIW and HSW. Figure 8 shows the shape and size of PAM_3.5%_/Cs and PAM_9%_/Cs before and after swelling in DIW for 24 h. Figure 9 depicts the profiles of swelling ratios in DIW and HSW to time (day), where all the PPGs reach their maximum swelling capacity in less than 1 h. In addition, one can notice that the highest swelling ratio was recorded for PAM_9%_/Cs with a value of 39.03 g/g and the lowest ratio for PAM_3.5%_/Cs with a value of just 4.65 g/g, suggesting that the highest PAM (polymer) content has the highest water holding capacity. The storage modulus results in DIW were opposite (Figure 10), as the most increased storage modulus was recorded for PAM_3.5%_/Cs at 5706.7 Pa and the lowest for PAM_9%_/Cs at 2583.8 Pa. Other PPGs, such as PAM_5%_/Cs, PAM_6.5%_/Cs, and PAM_8%_/Cs, have swelling capacities in the range of 18.05–37.74 g/g and storage moduli of 2706.9–3932.1 Pa in DIW. Bai et al. explained in their paper the reason for the increased swelling capacity of PPG with a higher amount of polyacrylamide, which is mainly related to the hydrophilicity of the amide groups (–CONH–) in PAM [13]. One might propose that the optimum concentrations of PAM with 0.5 wt% of chitosan are in the range of 5–9 wt%, as these concentrations can produce PPGs with high swellability and sufficient strength, given that the PAM_3.5%_/Cs PPG has poor swellability despite its high strength.

#### 3.3.2. Salinity Effect

Although PPGs are believed to be stable in saline formation water and the majority of reservoir mineralogies [7], unlike in-situ gels, it is still essential to understand their physical and mechanical characteristics in different saline media. Accordingly, four PPG samples of PAM/Cs out of nine are chosen to examine their swelling and mechanical strength behaviors in different saline media of 1% NaCl, SW, and HSW and to compare their attitude with that in DIW. As shown in Table 2, the swelling capacities of PAM_9%_/Cs_0.5%_, PAM_6.5%_/Cs_0.5%_, and PAM_6.5%_/Cs_0.25%_ PPGs in DIW are much higher than the swelling in brine solutions. For instance, the swelling ratio of PAM_6.5%_/Cs_0.25%_ was 80.37 g/g in DIW and dropped to 18.73 in HSW, mainly related to the concentration or osmotic pressure gradient, salt shielding effect, and electrostatic repulsion [13,18,38]. When comparing the swelling ratios of PAM/Cs PPGs in 1% NaCl, SW, and HSW, there is no noticeable difference, which can be attributable to the charge balance.

The swelling phenomenon of the PPG is a result of the osmotic pressure gradient across the PPG membrane, where the water molecules diffuse from the solution of lower osmotic pressure to the other side of higher osmotic pressure through the PPG membrane [39]. However, the presence of salts in the swelling medium increases their concentration and thus reduces the osmotic pressure difference across the PPG membrane, which is the driving force of absorbency, decreasing the PPG’s swellability [38].

**Figure 11:**
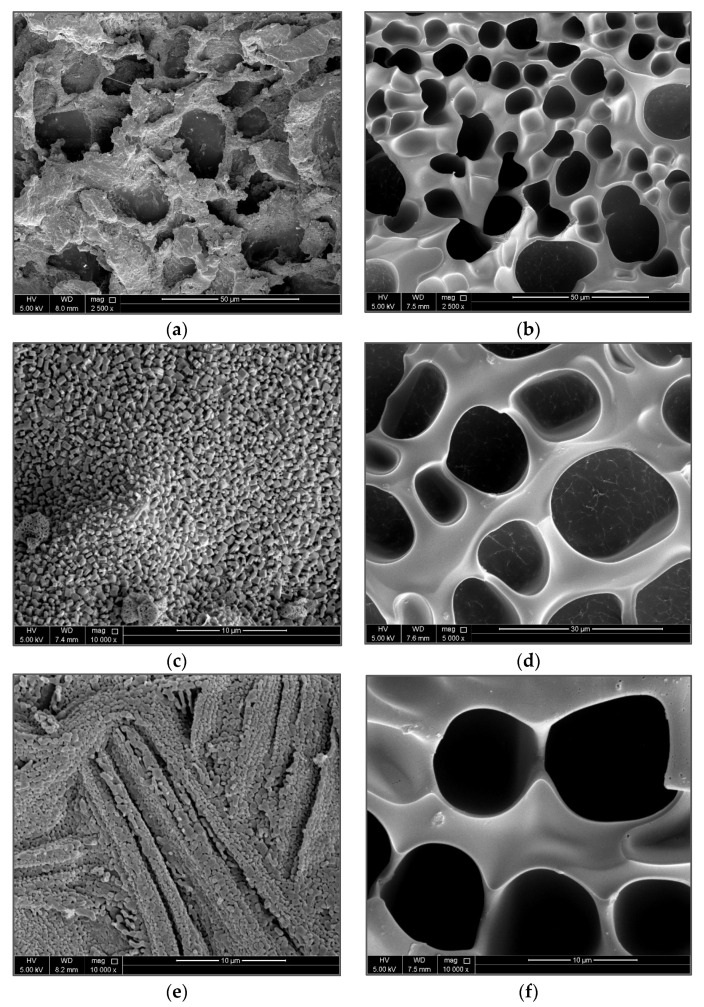
SEM images of different magnifications for PAM_3.5%_/Cs_0.5%_ in (**b**,**d**,**f**) distilled water; and (**a**,**c**,**e**) high saline solution.

Other studies [13,14,18,31] have explained that in the case of pure water, when no positive ions are present in the swelling medium, the hydrophilic groups in the PPGs will cause a powerful electrostatic repulsion force in the molecular structure. Thus, the hydrophilic groups generate chain spacing for water to enter and be absorbed, increasing the swelling capacity. In contrast, the presence of salts in the solution increases the positive ions (e.g., Na^+^, Ca^2+^, and Mg^2+^), which interact with the negatively charged groups (e.g., −COO^−^) in the PPGs’ chains to form ionic crosslinking and a shielding effect that reduces electrostatic repulsion, resulting in the shrinkage of chains and a decrease in the swelling ability (Figure 11e). The swelling ratios of different brine solutions are almost unchanged because all positive ions have reacted with the negatively charged groups, reaching a charge balance state where any further increase in salinity will not affect the swelling capacity.

Back to Table 2, PAM_3.5%_/Cs_0.5%_ showed different behavior, where the swelling ratio in DIW is lower than those in brine solutions by approximately 7 g/g. This action can be explained by the accumulation and adsorption of salt ions in the PPG surface, which increases the swelling ratio calculated by the gravimetric method. This fact can be seen in many studies [40], where chitosan acts as an adsorbent for heavy metal ions due to its adhesive feature [41]. In addition, SEM imaging (Figure 11) supports this argument, which clearly shows the accumulated salt particles on the PPG surface and inside its pores.

**Figure 12:**
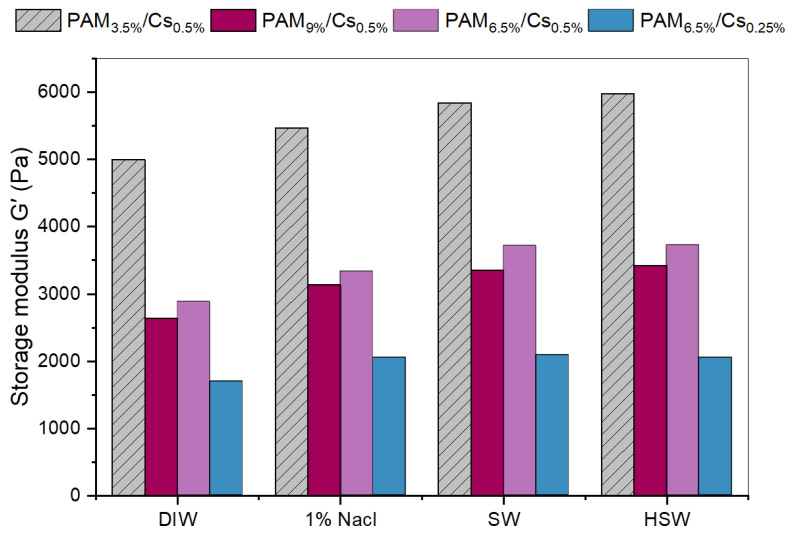
Storage modulus (G’) of swollen PAM/Cs in different salinity media.

Figure 12 depicts the storage modulus in different brine solutions and the DIW, which reflect the solid-like behavior and strengths of the gels. As expected, the trend of G’ was linearly increasing with the increment of salt amount, where the G’ was the highest in HSW and the lowest in DIW. This behavior is related to the interaction between the cations in the salts and negatively charged groups in the polymer gels that create ionic crosslinking and complexes, as seen in Figure 11a, which stiffened the PPG network structure [42,43]. Therefore, increasing the mechanical strength.

When comparing the behavior of PAM/Cs PPG in saline conditions with other PPG systems in the literature [17,23,25,42], one can notice their good behavior in terms of swelling capacity and ultimate strength, besides their non-toxicity property. In HSW, the TDS of 67.2976 g/L, the swelling ratios of PAM/Cs vary from 12.22 to 18.73 g/g, and the strengths are from 2052.9 to 5980.2 Pa.

#### 3.3.3. Effect of Temperature

Due to the diversity of the reservoir’s temperature, studying the maximum swelling ability and viscoelasticity of developed PPGs under the effect of temperature is imperative. Therefore, to investigate the temperature effect, known weights of each PPG sample were dispersed into HSW, sealed in screw-cap bottles, and submerged in an oil bath for 24 h at 25, 50, 75, and 100 °C. Figure S1A in the Supplementary Materials, illustrates the effect of temperature on the equilibrium swelling ratio of the PAM/Cs PPGs. The results showed that the swelling ratios of three PPGs of PAM_3.5%_/Cs_0.5%_, PAM_6.5%_/Cs_0.5%_, and PAM_9%_/Cs_0.5%_ had increased slightly with temperature increase from 25 to 100 °C, with an average increase in 1.16 g/g. In contrast, the swelling ratio of PAM_6.5%_/Cs_0.25%_ exhibited a minor decrease at 100 °C compared with 25 °C by 0.71 g/g. These actions are due mainly to the bonding expansion of the particle gel structures and increased water accessibility with temperature rise due to the thermal hydrolysis of amino groups (–CONH_2_) into acidic carboxylic groups (–COOH), which in turn increases the swelling capacity [13]. However, increasing the temperature and the thermal hydrolysis beyond the optimum conditions will open up more of the 3D network structure, leading to losses in the polymer chain’s connections and, thus, less water loss or syneresis and a lower swelling ratio [14]. Contrary to others, PAM_6.5%_/Cs_0.25%_ is most susceptible to polymer chain ruptures and syneresis due to lower crosslinker amounts. However, based on the results, all the PPGs have preserved their structures and swelling abilities even up to 100 °C.

The results of the storage modulus of the PAM/Cs PPGs versus various temperatures have been summarized in Figure S1B (Supplementary Materials) to support the justification of the swelling behavior mentioned earlier. As expected, the storage modules, which represent the ultimate strength of the particle gels, have marginally decreased as the temperature has risen. This decrease is primarily related to the network expansion and more water absorption into the particle gels, which enhance the swelling capacity and reduce the strength. The storage modules of PAM_3.5%_/Cs_0.5%_, PAM_6.5%_/Cs_0.5%_, PAM_9%_/Cs_0.5%_, and PAM_6.5%_/Cs_0.25%_ were 5980.20, 3725.7, 3425.8, and 2052.9 Pa, respectively, at 25 °C, while decreasing to 3344.3, 2569, 2559.6, and 1274.3 Pa, respectively, at 100 °C. All the PPGs’ strengths at 100 °C are still adequate to resist the reservoir’s continuous thermal and mechanical stresses. Accordingly, PAM/Cs PPGs have good thermal stability and are suitable for high-temperature reservoirs.

#### 3.3.4. Effect of pH

The pH is another factor that should be considered when designing a polymeric gel system, as some reservoir environments are highly acidic. This part of the investigation was accomplished in various swelling mediums of HSW with adjusted pH conditions, ranging from 2.0 to 12.0, by adding some droplets of dilute aqueous NaOH or HCl. Figure S2A, in the Supplementary Materials, shows the swelling ratios of PAM/Cs PPGs versus various pH (2, 4, 6, 8, 10, and 12). Although the trends were close to being confusing, two distinctive peaks of swelling ratio shifts were seen along the lines. These two peaks are found at pH = 4 and pH ≥ 10, mainly attributed to the high repulsion of –NH_3_^+^ groups in acidic media and –COO^−^ groups in basic media. Therefore, under acidic conditions, the amine groups in chitosan are protonated to ammonium cations (–NH_3_^+^), converting chitosan into a water-soluble cationic polyelectrolyte [44]. The formation of many positively charged ammonium groups creates electrostatic repulsions that expand the 3D network structure of the PPGs. They also increase the charge density inside the gel particles, which raises the osmotic pressure differential and induces water to transfer into the gel particles. Thus, increasing the swelling capacity. When the pH approaches 6, the amine groups undergo deprotonation, lowering the chitosan charges and making them insoluble. Therefore, particle gel networks will shrink and lose some of their potential to swell. Such behavior has been reported for superabsorbent hydrogels of chitosan-g-polyacrylamide [38] and poly(acrylic acid-co-acrylamide) grafted chitosan [28]. At alkaline conditions, the amide groups (–CONH_2_) in the polyacrylamide undergo basic hydrolysis into carboxylate groups (−COO^−^), enhancing electrostatic repulsions and water uptake capacity. Rani et al. have reported a similar observation of amide hydrolysis in the case of agar biosorbents [45].

Figure S2B (Supplementary Materials) illustrates the storage modulus (G′) of PAM/Cs PPGs in the pH range of 2–12. The results showed that the storage modules had a higher in pH = 6. This finding supports the previous mechanism of swelling behavior and chitosan’s natural properties. For instance, PAM_6.5%_/Cs_0.5%_ recorded a storage modulus of 2465.90, 3597.90, and 2387.90 Pa at pH of 2, 6, and 12, respectively. This behavior is aligned with the low swelling capacities at neutral conditions and the shrinkage of the PPGs network structures. In addition, hydrogen bonds may form between amines and carboxylic acids that cause some sort of crosslinking, leading to higher strengths [28].

### 3.4. Aging Effect Evaluation

**Figure 13:**
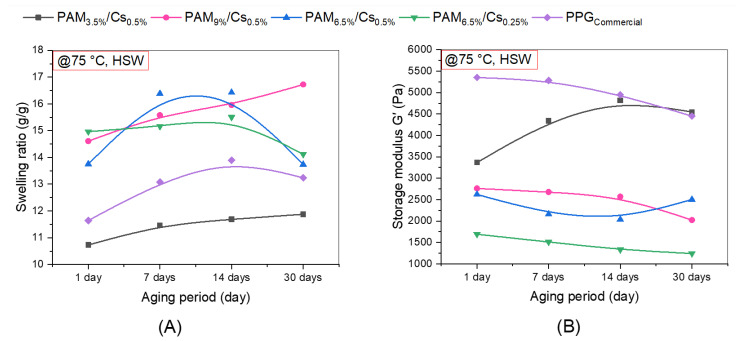
Aging effect on the (**A**) swelling and (**B**) strength of PAM/Cs PPGs and a commercial PPG at 75 °C in HSW.

According to Bai et al., PPG is deemed to have attained thermal stability when it maintains 80% of its initial strength at the given temperature [13]. In addition to ongoing high salinity effect and temperature effect experiments, this study section also considers the passage of time. Accordingly, the PPGs of PAM/Cs were placed in high saline solutions (TDS = 67.2976 g/L) and subjected to a constant temperature of 75 °C for 1 day, 7 days, 14 days, and 30 days to determine how long it would take to degrade. According to Figure 13A, the swelling ratios of PAM_3.5%_/Cs_0.5%_ and PAM_9%_/Cs_0.5%_ have increased on day 30 compared with day 1 by 10.66% and 14.48%, respectively. The cogent reason for such behaviors could be the expansion of the structure with continuous exposure to high temperatures for a period, which raises the water uptake. On the other hand, the swelling of PAM_6.5%_/Cs_0.25%_ witnessed an increase in the first 14 days by 3.59% and then exhibited a decrease on day 30 by −5.67% relative to the original swelling capacity. This may be due to increased water uptake, followed by structural deterioration or syneresis brought on by temperature and the anionic content of HSW on day 30 [27].

Moving to the elasticity behavior, in Figure 13B, the elastic or storage modules (G’) of PAM_9%_/Cs_0.5%_ and PAM_6.5%_/Cs_0.25%_ were aligned with the swelling results. The G’ of PAM_6.5%_/Cs_0.25%_ exhibited a declining rate of −26.59% related to increased water absorbency at the first 14 days, followed by structural raptures after 1 month, whereas the G’ of PAM_9%_/Cs_0.5%_ showed a declining rate of −26.83% related to increased swelling after 1 month. The swelling ratio and the storage modulus of PAM_6.5%_/Cs_0.5%_ were nearly unchanged at day 30 compared with day 1. On the other hand, the G’ of PAM_3.5%_/Cs_0.5%_ has grown by 35.06% despite greater swelling capacity after 1 month of aging. A commercial PPG of copolymers of AM and acrylic acid (AA) crosslinked with MBA was used in this investigation to provide a benchmark for comparison with the developed PAM/Cs PPGs. The swelling capacity of the commercial PPG showed a progressive increment in the first 14 days and then decreased slightly on day 30, whereas the storage modulus had been reduced by −16.86%.

Comparing the four PPGs of PAM/Cs with commercial PPG, one can notice that PAM_3.5%_/Cs_0.5%_ can provide nearly the same properties as commercial PPG, having a swelling capacity and storage modulus in the range of 11–13 g/g and 3300–5300 Pa, respectively. However, PAM_3.5%_/Cs_0.5%_ provides higher thermal stability, as the commercial PPG showed a decrease in strength after 30 days. Other PAM/Cs PPGs provided higher swelling capacities of 14–17 g/g, and good strengths of 1300–2800 Pa. PAM/Cs might be proposed as an alternative, efficient, and green PPG that can offer comparable features to other commercialized PPGs.

### 3.5. Particle Size

Zhao et al. claim that to capture the particle in the throat adequately, the swollen gel particle size must be larger than the pore throat size [46]. The sizes of pore throats range from nano to micro-scale [47]. The particle size selection of the preformed particle gel is a piece of critical knowledge, specifically when dealing with heterogeneous reservoirs. For the reason that undersized particles might not have the adequate blocking capacity to fill the channels or migrate out during successive water injections, the oversized particles may cause injectivity issues by blocking low-permeability zones [48]. Therefore, improper particle size selection may result in ineffective water shut-off performance and possibly irreversible permeability damage [48]. Equation (2) will be used in this investigation to initially estimate the swollen particle size of the developed gels using the swelling ratio and the average particle size of the dry PPG (Table 3). The dry particle size was defined by separating the crushed particles into particles of similar size using various wire mesh sieves; this study considers a size of 250–500 µm. Based on the results, the average swollen particle sizes in the distilled water medium were between 0.63 and 1.62 mm. On the other hand, the particle sizes in the HSW medium were in the range of 0.86 to 1.00 mm, indicating smaller particle sizes in saline environments. According to Bai et al., swollen particles could penetrate the pore throat by 0.175 times their diameter [49]. For instance, swollen particles with sizes between 0.63 and 1.62 mm will pass through pore throats with diameters between 0.11 and 0.28 mm. Nevertheless, future filtration experiments are required to accurately define the optimum particle size relative to the pore throat.

## 4. Conclusions

This study successfully developed and examined a novel, green, biodegradable PPG made of a polyacrylamide base polymer and a chitosan crosslinker for water shut-off. The developed PPG characteristics were investigated using FTIR, SEM, swelling measurements, and rheological assessment techniques. This investigation shows that chitosan can compete in the market and replace current commercial crosslinkers. The following are the main conclusions drawn from this study:

- The FTIR study has demonstrated the interaction between the functional groups of PAM and chitosan, which came about as a result of hydrogen bonds forming between amide groups with hydroxyl groups and amide groups with amine groups.
- The PPGs of PAM/Cs showed rapid swelling kinetics, with swelling ratios in DIW ranging from 5 to 107 g/g and in HSW ranging from 7 to 21 g/g, while the storage modulus was in the range of 939.12 to 21,857 Pa in HSW and 584.74 to 6775.7 Pa in DIW, depending on the concentrations of both PAM and chitosan.
- The swelling capacity of the PAM/Cs was found to increase with: (1) higher PAM concentration, (2) lower chitosan concentration, (3) higher temperature due to the formation of carboxylate groups, (4) freshwater medium, and (5) acidic and basic environments due to electrostatic repulsions.
- The storage modulus of PAM/Cs was higher with: (1) higher chitosan concentration that forms too many dense 3D network structures and very small pore size, (2) lower PAM content, (3) salinity conditions, (4) neutral swelling medium of pH≈6 due to hydrogen bonding formation, and (5) lower temperature.
- PAM/Cs PPGs showed good thermal and hydrolytic stability in the long term when aged at 75 °C and in the high-ionic medium for 1 month, indicating their ability to compete in the PPG market.
- The average particle size of the swollen PPG was estimated to be between 0.63 and 1.62 mm in DIW and 0.86 and 1.00 mm in HSW, as it is crucial to be defined for achieving good water shut-off performance.

## Figures and Tables

**Table 1:** Composition of HSW and SW based on Gulf reservoirs [29,30].

Salts	HSW Concentration (g/L)	SW Concentration (g/L)
NaHCO_3_	0.2382	0.1191
Na_2_SO_4_	6.5754	3.2877
CaCl_2_·H_2_O	2.3945	1.1972
MgCl_2_·6H_2_O	18.0539	9.1269
NaCl	40.2738	20.1369
TDS	67.2976	33.6488

**Table 2:** Equilibrium swelling ratios of PAM/Cs in different salinity media.

	PAM_3.5%_/Cs_0.5%_	PAM_9%_/Cs_0.5%_	PAM_6.5%_/Cs_0.5%_	PAM_6.5%_/Cs_0.25%_
DIW (g/g)	4.750	39.03	23.34	80.37
1% Nacl (g/g)	10.09	13.22	12.62	16.66
SW (g/g)	9.756	13.36	12.48	14.97
HSW (g/g)	12.22	15.83	16.70	18.73

**Table 3:** Particle size estimation of the swelled PAM/Cs PPG in DIW and HSW.

			In DIW			In HSW		
#	PPG Formulation	Avg. Size of Dry PPG, d_0_ (µm)	SR (g/g)	Avg. Size of Swollen PPG, Dt (µm)	Avg. Size of Swollen PPG, Dt (mm)	SR (g/g)	Avg. Size of Swollen PPG, Dt (µm)	Avg. Size of Swollen PPG, Dt (mm)
1	PAM_3.5%_/Cs_0.5%_	375	4.75	630.51	0.63	12.22	863.69	0.86
2	PAM_9%_/Cs_0.5%_	375	39.03	1272.00	1.27	15.83	941.53	0.94
3	PAM_6.5%_/Cs_0.5%_	375	23.34	1071.74	1.07	16.70	958.60	0.96
4	PAM_6.5%_/Cs_0.25%_	375	80.37	1618.29	1.62	18.73	995.92	1.00

## Data Availability

The data presented in this study are available on request from the corresponding author.

## Supplementary Materials

The following supporting information can be downloaded at: https://www.mdpi.com/article/10.3390/polym15081961/s1, Figure S1: The (A) swelling ratio and (B) strength of PAM/Cs PPGs in the temperature range (25–100 °C) in HSW for 24 h.; Figure S2: The effect of pH on the (A) swelling ratio and (B) strength of PAM/Cs PPGs in HSW for 24 h.
