# Supplementary material for: Biodegradable Preformed Particle Gel (PPG) Made of Natural Chitosan Material for Water Shut-Off Application
Source: Polymers (Basel). 2023 Apr 20;15(8):1961. doi: 10.3390/polym15081961 (PMC10144966; doi:10.3390/polym15081961)
Supplement: Supplementary file 1 [file polymers-15-01961-s001.zip › polymers-2256451-supplementary.pdf]

Supplementary Materials: Biodegradable Preformed Particle Gel (PPG) Made of Natural Chitosan Material for Water Shut-Off Application

Reem Elaf [1], Ahmed Ben Ali [1], Mohammed Saad [1,2], Ibnelwaleed A. Hussein [1,2,*], Hassan Nimir [3] and Baojun Bai [4]

[1] Gas Processing Center, College of Engineering, Qatar University, Doha P.O. Box 2713, Qatar
[2] Department of Chemical Engineering, College of Engineering, Qatar University, Doha P.O. Box 2713, Qatar
[3] Department of Chemistry and Earth Sciences, College of Arts and Science, Qatar University, P.O. Box 2713, Doha, Qatar;
[4] Department of Geosciences and Geological and Petroleum Engineering, Missouri University of Science and Technology, Rolla, MO 65409, USA
* Correspondence: ihussein@qu.edu.qa; Tel.: +974-4403-7694

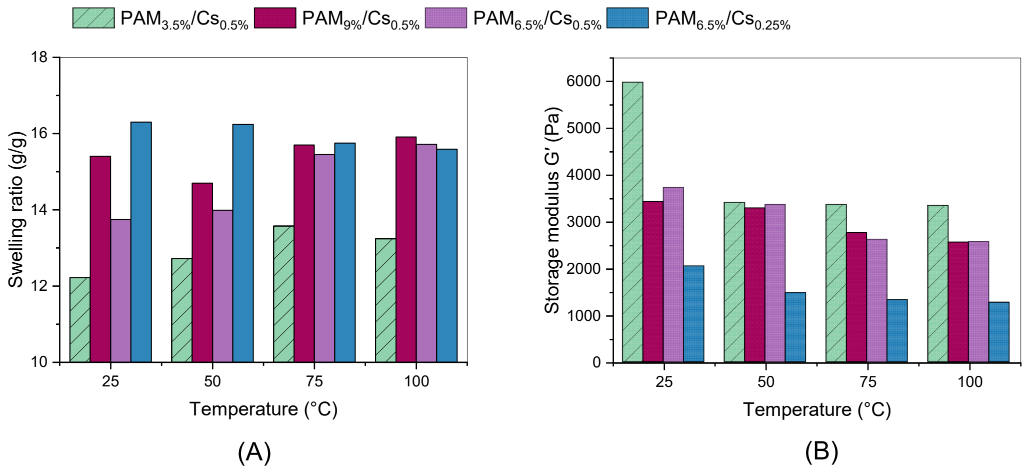

Figure S1. The (A) swelling ratio and (B) strength of PAM/Cs PPGs in the temperature range (25-100 °C) in HSW for 24 hours.

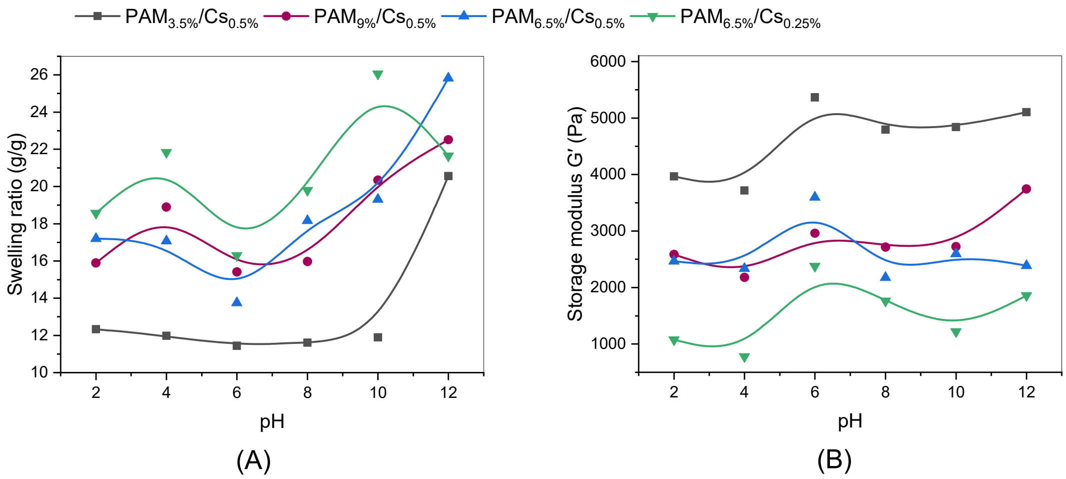

Figure S2. The effect of pH on the (A) swelling ratio and (B) strength of PAM/Cs PPGs in HSW for 24 hours.
